# Magnetic resonance thermal imaging combined with SMASH navigators in the presence of motion

**DOI:** 10.1120/jacmp.v13i4.3792

**Published:** 2012-07-05

**Authors:** Youngseob Seo, Jacob Willig‐Onwuachi, Jeffrey H. Walton

**Affiliations:** ^1^ Department of Radiology University of Texas Southwestern Medical Center at Dallas Dallas TX 75390; ^2^ Department of Biomedical Engineering University of California Davis CA 95616; ^3^ Department of Physics Grinnell College Grinnell IA 50112; ^4^ NMR Facility and Biomedical Engineering Graduate Group University of California Davis, Davis CA 95616 USA

**Keywords:** MR temperature imaging, PRF shift thermometry, SMASH navigators, liver motion, motion correction

## Abstract

This study develops and tests an MR thermometry method combined with SMASH navigators in phantom experiments mimicking human liver motion with the purpose of detecting and correcting motion artifacts in thermal MR images. Experimental data were acquired on a 3T MRI scanner. Motion artifacts of mobile phantoms mimicking human liver motion were detected and corrected using the SMASH navigators and then MR temperature maps were obtained using a proton resonant frequency (PRF) shift method with complex image subtraction. Temperature acquired by MR thermal imaging was compared to that measured via thermocouples. MR thermal imaging combined with the SMASH navigator technique resulted in accurate temperature maps of the mobile phantoms compared to temperatures measured using the thermocouples. The differences between the obtained and measured temperatures varied from 8.2°C to 14.2°C and 2.2°C to 4.9°C without and with motion correction, respectively. Motion correction improved the temperature acquired by MR thermal imaging by >55%. The combination of the MR thermal imaging and SMASH navigator technique will enable monitoring and controlling heat distribution and temperature change in tissues during thermal therapies and will be a very important tool for cancer treatment in mobile organs.

PACS number: 87.57.‐s

## I. INTRODUCTION

Over the last decade, minimally invasive thermal tumor ablation techniques may be an alternative to surgery or an adjuvant to other treatment modalities like chemotherapy and radiation therapy because they minimize incision size and reduce a patient's recovery time. Moreover, thermal ablation offers an alternative for patients who have nonresectable liver lesions. Ablation is achieved via local heating using ultrasound,^(^ ^1^ ^–^ ^4^ ^)^ laser,^(^ ^5^ ^,^ ^6^ ^)^ radiofrequency (RF),^(^ ^7^ ^–^ ^12^ ^)^ or microwave.^(^ ^13^ ^,^ ^14^ ^)^ Thus, effective treatment requires accurate temperature measurement of tumor tissues and the ability to monitor temperature change and heat distribution around the tumors. Magnetic resonance imaging (MRI)‐guided thermal therapy provides real‐time feedback, allowing for visualization and control of the thermal therapy and preventing normal tissues surrounding the tumors from being damaged.^(^ ^15^ ^–^ ^18^ ^)^

However, the motion of internal mobile organs represents a major challenge for the MRI‐guided thermal therapy because MR temperature maps are easily corrupted by motion.^(^ ^19^ ^–^ ^21^ ^)^ For a mobile organ, such as a liver, respiratory motion causes the blurring and ghosting of MR images. Moreover, respiration leads to erroneous MR temperature mapping because temperature changes are derived from changes in MR image phases^(^ ^22^ ^)^ and if the underlying tissue moves, the pixel phase may change regardless of temperature.^(^ ^23^ ^)^

Previous studies have proposed different approaches to reduce motion artifacts. The combination of SMASH^(^ ^24^ ^)^ with navigator echoes was used to detect and correct the motion artifacts.^(^ ^25^ ^)^ The motion‐corrupted navigator echoes were replaced by those acquired from the SMASH technique. In other studies with respiratory‐ or velocity‐triggered pulse sequences, navigators were incorporated in detection and correction of breathing motion artifacts to obtain MR temperature maps.^(^ ^26^ ^–^ ^28^ ^)^ The first navigator echo acquired prior to heating was used as the reference profile and was compared to data acquired during heating. The phase difference between subsequent navigator echo and the reference was detected, and then the correction of phase shifts derived from respiratory motion was performed in k‐space.

The purpose of this study is to develop and test an MR thermometry method combined with the SMASH navigator technique^(^ ^25^ ^)^ to detect and correct motion artifacts in phantom experiments mimicking human liver motion. We will combine the SMASH navigator technique for detection and correction with complex subtraction proton resonance frequency (PRF) shift^(^ ^29^ ^)^ thermometry method to demonstrate the availability of MR thermal imaging in the presence of motion.

## II. MATERIALS AND METHODS

### A. MR systems

Two distinct MRI systems were employed. A Bruker 7T MRI system (Bruker Biospin MRI Inc., Billerica, MA, USA) for small animal imaging was used for preliminary studies to develop our thermal imaging capabilities. The two‐dimensional multislice images were acquired using fast low angle shot (FLASH) with parameters: TR=33 ms, 6 slices, matrix size =128×128, field of view $(\text{FOV})=5.5\;{\text{cm}}^{2}$, $\text{voxel}=0.4\times 0.4\times 3.0\;{\text{mm}}^{3}$, bandwidth (BW)=390 Hz/pixel, and angle=20°. Even though an echo time (TE) equal to $T_{2}^{*}$ of the tissue of interest is generally optimal for the PRF shift thermometry,^(^ ^30^ ^)^ short TE of 3.26 ms was chosen in this study. Our previous work has demonstrated that a short TE (= 3.26 ms) FLASH sequence was reliable for obtaining temperature maps of fried chicken fingers and gel phantoms using the PRF shift method.^(^ ^31^ ^,^ ^32^ ^)^ The MRI data acquisition time was 3.2 sec.

A Siemens 3T Trio Tim whole‐body MRI system (Siemens Medical Solution, Erlangen, Germany) was employed to acquire experimental data. Data was taken with an 8‐channel head coil array. Data from each of the eight component coil were obtained simultaneously and stored separately for postprocessing. The used MRI sequence was FLASH with parameters: TE=3.6 ms, TR=14 ms, 3 slices, matrix size = 64 ×256×8 (reconstructed to 128×128×8), $\text{FOV}=12.8\times 12.8\;{\text{cm}}^{2}$, $\text{voxel}\;=1.0\;\times 1.0\times 6.0\;{\text{mm}}^{3}$, BW=1000 Hz/pixel, and flip angle =10°. The total acquisition time was 3 sec.

### B. Phantom preparation

**Figure 1:**
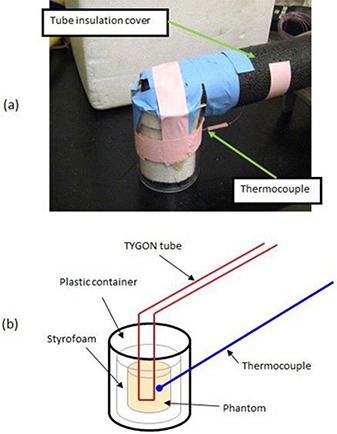
Assembled phantom is shown in (a) and a schematic drawing of the arrangement is in (b). The gel phantom with Styrofoam insulation is placed in a transparent plastic container. A thermocouple is inserted into the phantom to measure real temperatures. A TYGON tube passes through the phantom to continuously deliver hot water.

An organic hydrophilic polymer gel phantom (4 cm diameter by 4 cm high) was made by mixing 230 g of TX 151 solidifying powder (Oil Center Research Intl. L.L.C., Lafayette, LA, USA) with 23 g of deionized water at 4°C. The solution was mixed slowly in a 1000 mL beaker. When the powder was evenly dispersed, a vacuum was pulled on the beaker to eliminate air bubbles in the mixture. The mixture was poured into 50 mL beakers and then heated in a water bath at 80°C for 1 hour until the solution looked firm. The beakers were covered with plastic and the mixture turned to a gel at room temperature for at least 24 hrs. The gel phantoms were cut to fit into a Styrofoam holder (4 cm inner diameter ×4 cm high ×2 cm thick) to prevent heat loss, and then the Styrofoam holder was inserted into a transparent plastic container (6 cm inner diameter ×8 cm high ×1 cm thick) to prevent leakage of heating fluid or the gel in case the phantom tipped over (Fig. 1). The plastic container was placed in the wood platform (10 cm × 17 cm rectangle) to slide smoothly back and forth. The gel thermal properties were measured with a thermal property analyzer (KD2, Decagon Devices Inc., Pullman, WA, USA). Shear stiffness was also measured.^(^ ^33^ ^)^

A single continuous TYGON tube (Fisher Scientific Inc., Pittsburgh, PA, USA) was imbedded in the phantom and delivered 60°C water. The tube (0.16 cm inner diameter ×0.31 cm outer diameter ×15 m long) was covered by standard plumbing insulation materials to prevent heat loss during transit. A 20 meter long type T thermocouple was embedded in the gel very close to the tube to measure the real temperature while hot water was flowing through the tube. The distance between the tip of the thermocouple and the tube was < 2 mm. Then the measured thermocouple temperature was compared to that obtained by MR thermal imaging.

### C. Phantom motion

The dominant component of motion in the human liver motion is cranial–caudal translation.^(^ ^34^ ^)^ The magnitude of this translation between inhalation and exhalation ranged from 1.2 to 2.6 cm. Anterior–posterior translation was between 0.1 and 1.2 cm, while motion in the left–right direction was between 0.1 and 0.3 cm. Rotation did not exceed 1.5°. Korin et al.^(^ ^35^ ^)^ assumed that motion of the liver had an average amplitude of 1.3 cm during normal breathing. The period of one cycle of a normal human respiration between inhalation and exhalation is 4 sec.^(^ ^36^ ^)^ Thus, a simple approximation of the liver respiratory motion is a linear shift followed by a return to the original position without deformation and rotation. The phantom was moved in a controlled fashion during scanning to simulate a respiratory‐like motion. We simulated *in vivo* liver motion as simple, linear harmonic motion with amplitude of 1.5 cm and frequency of 0.25Hz. This motion was generated using a MD‐2 dual stepper motor system (Arrick Robotics Inc., Tyler, TX, USA). The stepper motor was located inside the magnet room and continuously rotated an aluminum disk at a constant angular speed of 1.56 rad/sec. The disk was connected off‐center to a flexible nonmagnetic rod (4.6 m long). The rotating disk drove the rod back and forth connecting the disk to the phantom carrier, transforming a rotational motion into a linear motion.

### D. Heating device

Two different heating devices were employed. For the 7T study, a GE microwave oven (GE Inc., Louisville, KY, model No. JT930BHBB) was used to heat the phantoms. The heating times were 2 min, 6 min, and 8 min. The air was heated by heating elements placed at the top, bottom, and the back wall, while the air was circulated by a fan at the back of the oven using convection mode. The gel phantoms were imaged at room temperature, heated in the GE oven and returned to the magnet for subsequent thermal imaging. The phantoms could be returned to the same position in the magnet with less than a 100 micron error using a sample holder built for this purpose.

For the 3T study, a water bath (Fisher Scientific Inc., Versa‐bath, Pittsburgh, PA) was employed to maintain constant 60°C water. A diaphragm pump (Liquid Metronics Inc., Garden grove, CA) was mounted on the water bath. The pump was used to continuously push the hot water from the water bath through the tube. After the water traversed through the phantom, it was returned to the water bath.

### E. Complex subtraction PRF shift thermometry

In MRI, phase discontinuities arise from the fact that the phase is defined only in the range of (−π,π). An individual phase image has wrapped phases less than −π or greater than π, resulting in phase discontinuities between adjacent pixels. This problem with the phase discontinuity is compounded when two phase images are subtracted. In order to prevent the phase discontinuity problem between the adjacent pixels, the complex subtraction method was used.^(^ ^37^ ^,^ ^38^ ^)^ Briefly, the complex image of the reference is multipled by the complex conjugate of the thermal image pixel by pixel. The arctangent of the resulting image yields the phase difference, Δφ. Then the temperature was calculated fom:^(^ ^29^ ^)^

(1) $$\Delta T=\frac{\Delta \varphi }{\gamma \alpha B_{0}TE}$$

where ΔT=temperature difference, Δφ=phase difference, α=PRF change coefficient for aqueous tissue (−0.01ppm/°C), γ=gyromagnetic ratio, $B_{0}=\text{main}\;\text{magnetic}\;\text{field}$, and TE=echo time. Phase images acquired by complex subtraction were compared to those obtained by direct phase subtraction. The starting temperature of the gel was measured as the reference temperature using the thermocouple.

### F. Data and image processing

MRI datasets were postprocessed using a workstation (Dual‐core Intel Xeon 5200 processor with 1.86GHz) running custom software written in MATLAB (The MathWorks, Natick, MA).

First, detection and correction of the motion artifacts of the mobile phantom without heating was performed using SMASH navigators. A Levenberg‐Marquardt algorithm^(^ ^39^ ^,^ ^40^ ^)^ with a 95% confidence level was used to detect phase difference between the measured navigator echo and the predicted echo (as predicted by the previous echo) using the measured coil sensitivities. Magnitude images of each component coil of the eight‐channel head coil array were acquired. Using each magnitude image, the square root of the sum of the squares of each coil image (SOS image) was obtained as follows:

(2) $$\text{SOS}\;\text{image}=\sqrt{\sum_{k=1}^{N}{\left(I_{k}\right)}^{2}}$$

where $I_{k}$ is each coil image and *N* is the number of component array coils.

The coil sensitivities were acquired by dividing each coil image by the SOS image. Then negative, zero, and positive harmonic weights for SMASH fittings were obtained by using the coil sensitivities. The coil sensitivities, $C_{k}(x,y)$, are multiplied by appropriate linear weights, $w_{k}^{m}$, to generate composite sensitivity profiles, $C_{m}^{comp}(x,y)$, with sinusoidal spatial sensitivity variation of the order m:^(^ ^24^ ^)^

(3) $$C_{m}^{comp}(x,y)=\sum_{k=1}w_{k}^{m}\times C_{k}(x,y)≅\text{exp}({im\Delta k}_{y}y)$$

where m= the order of the generated spatial harmonic (an integer), k= number of element array coils, and $\Delta k_{y}=2\pi /\text{FOV}$. Per SMASH navigators procedure, phase encoding (PE) line 1 in a k‐space domain was assumed to be not corrupted by motion. Using the PE line 1, we predicted PE line 2 and compared it to the measured PE line 2. If there was difference in phase values between the predicted and measured lines, the measured line was replaced by the predicted one and we jumped to next PE line, and this continues through all PE lines.

Secondly, MR thermal imaging of static phantoms was implemented using the complex subtraction PRF shift thermometry. In order to assess the accuracy of the temperature obtained using the MR thermal imaging, 9 pixels were selected above the thermocouple and an average value was taken. The temperature acquired by the thermal imaging technique was compared to the real temperature measured via the thermocouple.

Finally, MR thermal imaging in combination with the SMASH navigator technique of moving phantoms was performed. The SMASH navigator technique was implemented and followed by the complex subtraction PRF shift temperature imaging to obtain temperature maps of the mobile phantoms. The time needed for the postprocessing to obtain temperature maps was < 10 sec.

### G. Comparison of signal‐to‐noise ratio (SNR) before vs. after motion

SNR was calculated in a single magnitude image using software written in MATLAB. The signal (S) was measured as a mean intensity in a region‐of‐interest (ROI) of 10× 10 pixels with the maximum uniform signal inside the phantom. The noise (a) was evaluated as a standard deviation of pixel intensity in the same size ROI located in the background. SNR was computed with:^(^ ^41^ ^)^

(4) $$SNR=\frac{S}{\sigma }$$

### H. Comparison of Mr temperature maps of a mobile phantom with vs. without motion correction

MR temperature maps of the moving phantom with motion correction using the SMASH navigator technique were compared to those without the correction. The percentage improvement of temperatures the motion correction made was calculated by (1−ω)×100, where ω= (percent difference between the measured and obtained temperatures with motion correction) / (percent difference between the measured and obtained temperatures without motion correction).

## III. RESULTS

### A. Physical properties of gel phantoms

Table 1 shows a comparison of thermal properties of the phantoms with those of a human liver. Note that they are within 10% of each other. Furthermore, the shear stiffness of the human liver is 2.0±0.3 kPa^(^ ^42^ ^)^ and the shear stiffness of the phantom was 1.5 kPa. Thus the gel phantom has thermal and mechanical properties very similar to the human liver.

**Table 1:** Thermal properties of TX151 gel phantoms and human liver compared.

	*TX 151 Gel Phantom*	*Human Liver* ^(^ ^46^ ^,^ ^47^ ^)^
*k* [W/(m·K)]	0.537±0.003	0.467–0.527
$C_{p}[J/(\text{kg}\cdot K)]$	5475±482	4814–5296
$\rho [\text{kg}/m^{3}]$	990±20	995–1015

### B. Comparison of complex subtraction to direct phase subtraction method

**Figure 2:**
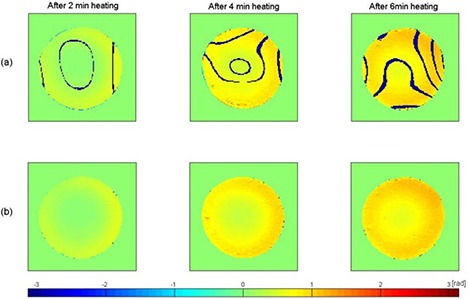
Phase images are obtained by direct pixel‐by‐pixel phase subtraction in row (a) and by complex subtraction in row (b). They have same color scale [‐π, π]. Dark blue stripes in row (a) are due to phase discontinuities.

**Figure 3:**
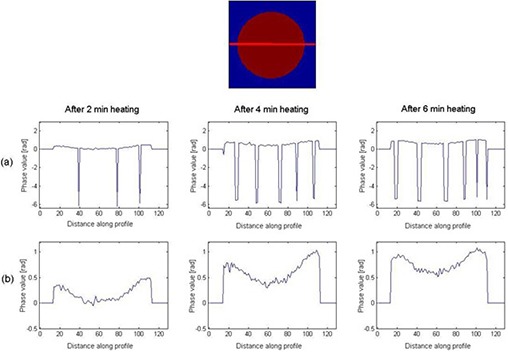
Phase values in the centerline of phase images are obtained by direct pixel‐by‐pixel phase subtraction in row (a) and complex subtraction in row (b). The vertical axis is phase and horizontal axis is the distance (pixel number) along profile. Row (a) displays phase discontinuities along the profile.

Figure 2 shows the phase images that were created from the raw data acquired at 7T by the direct phase subtraction and the complex subtraction. The direct pixel‐by‐pixel phase subtraction shows phase discontinuities. However, the complex subtraction indicates continuous, uniform phase distribution. Centerlines along the left to right direction from the phase images shown in Fig. 2 are plotted in Fig. 3. Phase discontinuities (<−π) are apparent in the centerline of the phase images obtained from the direct pixel‐by‐pixel phase subtraction, whereas they are absent in the centerline derived from the complex subtraction. Figure 3 clearly demonstrates that the complex subtraction method is better than the direct phase subtraction method.

### C. Detection and correction of motion artifacts without heating

**Figure 4:**
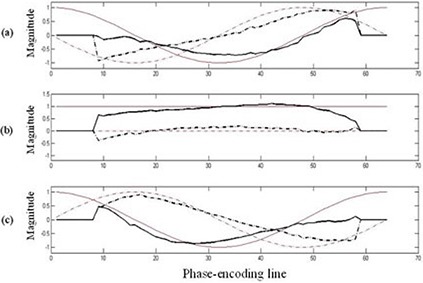
Comparison of spatial harmonic profiles obtained from coil sensitivities (black) vs. ideal target harmonic profiles (red). The harmonics used were negative first (a), zero (b), and positive first (c). The real part (solid line) and imaginary part (dashed line) of the complex harmonic profiles are shown.

Figure 4 plots the SMASH fittings for the negative first, zero, and positive first harmonics. The harmonic fits do not exactly match to the target spatial harmonics and this can result in errors when detecting and correcting the motion artifacts because SMASH reconstructions rely upon the accurate knowledge of the coil sensitivity of each component surface coil in the array.^(^ ^24^ ^)^

**Figure 5:**
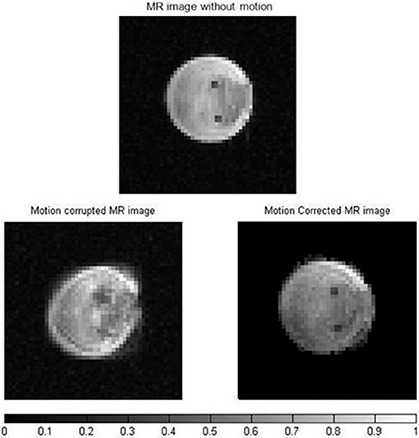
MR magnitude images of the phantom without motion (top), motion corrupted (left) and corrected (right). The imparted motion mimics that of liver during breathing. The color bar shows normalized signal intensity. There is a drop in signal after motion correction.

Motion corrupted and corrected images are shown in Fig. 5. The MATLAB code progressed line by line through the k‐space detecting and correcting each motion corrupted PE line. The motion corrupted image is blurred and the two small holes due to the TYGON tube are not easily seen. However, motion artifacts are removed in the corrected image and the two small holes are much clearer. The SNR values before and after the phantom motion were 66.1±4.2 and 40.7±3.3), respectively. The SNR dropped by 38.4% due to the motion.

### D. MR thermal imaging of a static phantom

**Figure 6:**
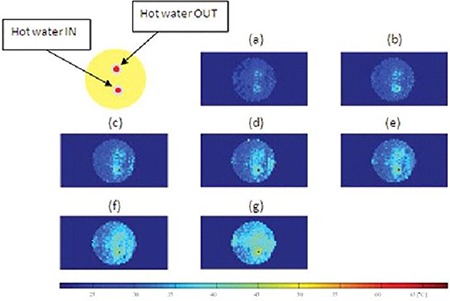
MR temperature maps of a static phantom at 10 min (a), 20 min (b), 30 min (c), 40 min (d), 50 min (e), 60 min (f), and 70 min after the start of heating. Temperature (°C) is given in the color bar.

Figure 6 shows MR temperature maps acquired on the 3T at 10 min (a), 20 min (b), 30 min (c), 40 min (d), 50 min (e), 60 min (f), and 70 min (g) after the water pump was turned on to continuously push hot water through the tube. The temperature maps were acquired by the complex subtraction PRF shift method. The temperature difference between the hot water IN and OUT was 8°C–10°C. Note that as heating time increases, temperature inside the phantom increases. After 10 min of heating, most of the phantom remains at room temperature (22°C), but the area around the input and output of hot water is at elevated temperature. The temperature around the input of hot water was at 33.6°C whereas actual temperature measured via the thermocouple was 35.5°C. At 70 min after the phantom was heated, the majority of the phantom has reached 43.5°C.

**Figure 7:**
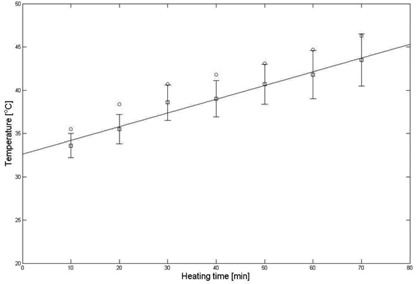
Comparison of temperatures (Fig. 6) obtained from a static phantom (black squares) by thermal imaging vs. those (red circles) measured via a thermocouple. The thermal imaging temperature is the average over 9 pixels around the location and the error bars on the thermal imaging are the standard deviation of the 9 pixels.

Table 2 shows temperatures obtained by the thermal imaging technique, temperatures measured via the thermocouple, and the difference between them. The temperature obtained using the MR temperature imaging is consistently lower than that measured via the thermocouple. The differences between the obtained and measured temperatures vary from 1.9°C to 2.9°C. Figure 7 shows a graph of temperature change of a static phantom according to heating times and a comparison of temperature obtained by the thermal imaging to that measured via the thermocouple. Linear polynomial curve fitting for the obtained data using the thermal imaging was implemented with 95% confidence and showed that the MR thermometry technique was linear with the static phantom material as a function of heating times. There is a systematic error of 2.5°C between the two methods.

**Table 2:** Temperatures of a static phantom obtained via MR thermal imaging and those measured via a thermocouple. MR thermal imaging temperatures are an average over 9 pixels and the error is± one standard deviation.

*Heating Time*	*Temperature (* $T_{MRTI}$ *) Obtained by MR Thermal Imaging (* °C *)*	*Temperature (* $T_{TC}$ *) Measured by a Thermocouple (* °C *) (* Error = ±0.1 *)*	*Temperature Difference (* $\Delta T=T_{TC}-T_{MRTI}$ *) (°C) (* Average=2.5 *)*
10 min	33.6 (±1.4)	35.5	1.9
20 min	35.5 (±1.7)	38.4	2.9
30 min	38.6 (±2.1)	40.7	2.1
40 min	39.0 (±2.1)	41.8	2.8
50 min	40.7 (±2.3)	43.1	2.4
60 min	41.8 (±2.8)	44.7	2.9
70 min	43.5 (±3.0)	46.3	2.8

### E. MR thermal imaging of a mobile phantom

**Figure 8:**
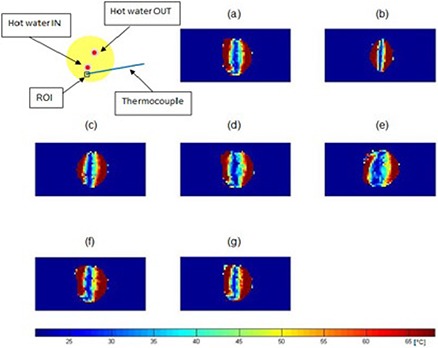
MR temperature maps of a mobile phantom without motion correction at 10 min (a), 20 min (b), 30 min (c), 40 min (d), 50 min (e), 60 min (f), and 70 min (g) after the start of heating. Temperature (°C) is given in the color bar. Note the large temperature distortions that result from not correcting the motion.

**Figure 9:**
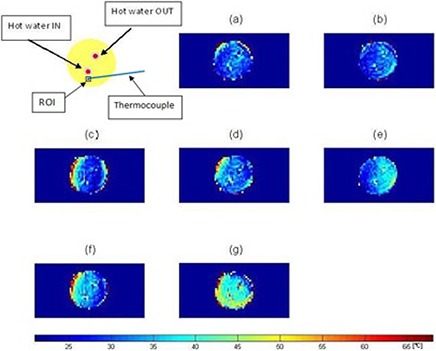
MR temperature maps of a mobile phantom with motion correction at 10 min (a), 20 min (b), 30 min (c), 40 min (d), 50 min (e), 60 min (f), and 70 min (g) after the start of heating. Temperature (°C) is given in the color bar.

The temperature maps of the moving phantom in the presence of motion at 10 min (a), 20 min (b), 30 min (c), 40 min (d), 50 min (e), 60 min (f), and 70 min (g) heating without and with motion correction are shown in Figs. 8 and 9, respectively. Figure 8 shows very heterogeneous temperature distribution: low temperature (=23°C) in the middle and high temperature (=69°C) in the periphery. Two hotter spots are not seen. However, Fig. 9 indicates much better uniform temperature distribution than Fig. 8. As heating time increases, temperature increases. In the temperature maps, there are the two hotter spots. One is the input of heating water and the other is the output.

Table 3 shows temperatures of a moving phantom without the motion correction acquired by the MR thermal imaging technique, temperatures measured via the thermocouple, and the difference between them. The temperatures obtained by the MR thermal imaging technique were lower than 36°C irrespective of heating times. The differences between the obtained and measured temperatures vary from 8.2°C to 14.2°C

**Table 3:** Temperatures of a mobile phantom without motion correction acquired by MR thermal imaging and those measured via a thermocouple. For the temperatures obtained by MR thermal imaging, an average value (± standard deviation) of 9 pixels is taken.

*Heating Time*	*Temperature (* $T_{MRTI}$ *) Obtained by MR Thermal Imaging (* °C *)*	*Temperature (* $T_{C}$) *Measured by a Thermocouple (* °C *) (* Error = ±0.1 *)*	*Temperature Difference (* $\Delta T=T_{TC}-T_{MRTI}$ *) (* °C *)* (Average=10.9)
10 min	28.9 (±5.1)	37.1	8.2
20 min	31.5 (±6.5)	39.9	8.4
30 min	30.2 (±6.6)	41.6	11.4
40 min	31.7 (±5.3)	42.7	11.0
50 min	29.8 (±7.2)	44.0	14.2
60 min	35.9 (±6.1)	45.8	9.9
70 min	34.4 (±6.2)	47.5	13.1

**Figure 10:**
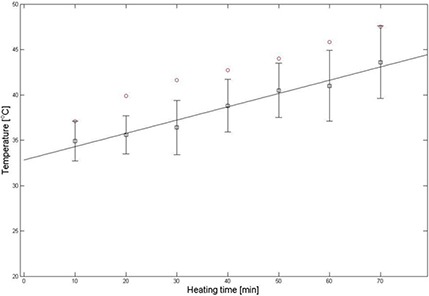
Comparison of temperatures of a motion‐corrected mobile phantom (black squares) acquired by thermal imaging with those (red circles) measured via a thermocouple. The ROI (a black square) is placed as indicated in Figure 9. The thermal imaging temperature is the average over 9 pixels around the location and the error bars on the thermal imaging are the standard deviation of the 9 pixels.

Table 4 indicates temperatures of the moving phantom with the motion correction obtained by the thermal imaging, temperatures measured via the thermocouple, and the difference between them. The differences between the obtained and measured temperatures range from 2.2°C to 4.9°C. Motion correction improved the temperature acquired by MR thermal imaging by >55%. Figure 10 represents a graph of temperature change of the mobile phantom according to the heating times, and a comparison of temperatures obtained by the thermal imaging to those measured via the thermocouple. Linear polynomial curve fitting for the data obtained using the thermal imaging was implemented with 95% confidence and showed that the MR thermometry technique combined with the SMASH navigator technique was linear with the mobile phantom material. There is a systematic difference of 3.8°C between the two methods.

**Table 4:** Temperatures of a mobile phantom with motion correction acquired by MR thermal imaging and those measured via a thermocouple. For the temperatures obtained by MR thermal imaging, an average value (± standard deviation) of 9 pixels is taken.

*Heating Time*	*Temperature (* $T_{MRTI}$ *(* *Obtained by MR Thermal Imaging (* °C *)*	*Temperature (* $T_{TC}$ *) Measured by a Thermocouple (* °C *) (* Error = ±0.1 *)*	*Temperature Difference (* $\Delta T=T_{TC}-T_{MRTI}$ *) (* °C *)* (Average=3.8)
10 min	34.9 (±2.2)	37.1	2.2
20 min	35.6 (±2.1)	39.9	4.3
30 min	36.7 (±3.0)	41.6	4.9
40 min	38.8 (±2.9)	42.7	3.9
50 min	40.5 (±3.0)	44.0	3.5
60 min	41.0 (±3.9)	45.8	4.8
70 min	43.6 (±4.1)	47.5	3.9

## IV. DISCUSSION

Liver motion modeling was investigated using MR imaging with prospective gating, but it is found that the gating created impractically long imaging times.^(^ ^43^ ^)^ Liver motion artifacts induced by respiratory movement have been reduced by using fast MR imaging techniques: echo‐planar imaging (EPI) combined with respiratory gating.^(^ ^44^ ^)^ This technique, however, does not provide coregistration among all time points, sacrifices SNR, spatial resolution, or temporal resolution, and has not always proven practical in human imaging. We are unaware of previous studies using thermal therapy combined with parallel MRI techniques in a mobile organ of either animals or humans.

The impacts of the stepper motor on image quality were SNR loss and motion artifacts. The motion artifacts of the phantom mimicking the human liver motion were effectively detected and corrected by the SMASH navigator technique. Even though the harmonic weights (the negative first, zero, and positive first) acquired by the coil sensitivities were not perfectly fit to the target harmonics, good MR images were obtained without ghosting and blurring.

MR temperature maps of the static phantom were obtained using the complex subtraction PRF shift method with a gradient echo sequence. The phantom was heated by 60°C water and thermal conduction. The hot water IN tube appeared in the image as a hot (red) spot and the hot water OUT tube showed up as a yellow spot because of a large temperature gradient between the 60°C water and the 22°C gel phantom. The temperature dropped quickly due to heat transfer between them. The contact length (=80 mm) of the tube to the phantom was long compared to 1.6 mm diameter and 1.5 mm thickness of the tube, causing radial heat conduction to sufficiently occur for a short time. The temperature profile consistently increased with the heating times. At 70 min after heating, the phantom has nearly uniform temperature distribution. Temperatures obtained by the MR thermal imaging were close to those measured via the thermocouple.

**Figure 11:**
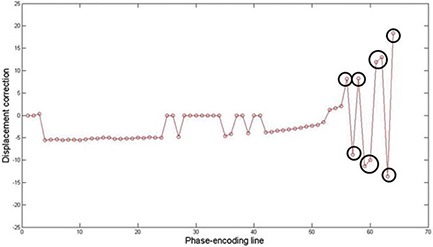
Displacement correction for each PE line in k‐space at 30 min after heating. Motion artifacts are corrected in all PE lines except 56–64 (black circles). Some motion‐corrupted PE lines are not corrected using SMASH navigator technique.

Temperature maps of the mobile phantom with the correction of the motion artifacts using the SMASH navigator technique were much better than those without the correction. The data is consistent with what is intuitively expected — the phantom temperature increases as the heating time increases. Furthermore, the areas of the input and output of hot water have higher temperature than the rest of the sample. However, the temperature distribution along the periphery of the phantom is still inconsistent, even though the proposed motion correction technique corrected the motion artifacts and significantly improved the temperature accuracy of the moving phantom. At times, motion corrupted PE lines were missed resulting in phase discontinuities. For example, the displacement correction for each PE line in the k‐space at 30 min after heating was plotted in Fig. 11. The motion artifacts were corrected in all PE lines except lines 56–64. The use of the imperfect harmonic weights obtained by the coil sensitivities led to poor estimation of pixel shift or phase change. This in turn resulted in poor motion correction or missed motion corrupted PE lines causing the error to be cumulative. This problem was caused by the fact that the negative first, zero, and positive first harmonic weights for the SMASH fittings obtained by the coil sensitivities were not perfectly identical to the ideal harmonics shown in Fig. 4.^(^ ^24^ ^,^ ^25^ ^)^ Thus, the temperature maps have uniform temperature distribution except at the periphery. The difference between temperatures obtained by the thermal imaging and those measured via the thermocouple with a systematic error of 3.8°C was bigger than that of the static phantom possibly because of the phase discontinuities and/or poor motion correction caused by the acquired imperfect harmonic weights.

The differences in temperature readings derived from the thermal imaging and thermocouples in both static and mobile phantoms can be explained by a spatial mismatch between the tip of the thermocouples and the ROI selected in the MR temperature images, magnetic field variations due to a redistribution of magnetic susceptibility caused by motion outside ROIs, or both.^(^ ^45^ ^)^

The limitation of this study is that the first PE line is assumed to be without motion‐corruption, based on the SMASH navigator technique. Otherwise, the motion‐corrupt first PE line may affect the rest of the PE lines and cause poor motion correction in all PE lines. Another limitation is that the phantom used in this study does not at all mimic the actual human liver anatomy, nor does it mimic the actual liver motion and deformation. The assumption that the liver motion may be modeled by a simple rigid translation only holds for the part of the liver below the rib cage. The motion of tissues in the upper part of the liver is much more complex. Furthermore, the FOV was large enough to contain the small phantoms, but too small to allow for abdominal imaging in a human subject. Changing the pulse sequence to accommodate for larger subjects will change its performance in terms of spatial resolution, temporal resolution, and SNR.^(^ ^20^ ^)^ However, the use of SMASH navigators for MR thermometry purposes may be a novel approach in a moving object.

## V. CONCLUSIONS

In conclusion, an MR thermometry method combined with the SMASH navigators in phantom experiments in the presence of motion was developed and tested. The combination of the MR thermal imaging and parallel MRI techniques will enable monitoring of the heat distribution and temperature change in tissues during thermal therapies, and will be a very important tool for cancer treatment in mobile organs.

## VI. ACKNOWLEDGEMENTS

The authors are grateful acknowledge the Imaging Research Center at University of California Medical Center for pilot grant funding for the use of the 3 Tesla MR system.
